# Escalating Mortality From Opioid-Related Deaths With Concurrent Gastrointestinal Complications in the United States, 2010–2020: A Population-Based Analysis

**DOI:** 10.7759/cureus.86489

**Published:** 2025-06-21

**Authors:** John K Appiah, Richeal Asante, Chukwunonso B Ubanatu, George S Blewusi, Ewurabena Plange-Kaye, Emmanuel K Asiedu

**Affiliations:** 1 Internal Medicine, Geisinger Health System, Wilkes-Barre, USA; 2 Public Health, Johns Hopkins Bloomberg School of Public Health, Baltimore, USA; 3 Dentistry, Columbia University, New York City, USA; 4 Internal Medicine, Mother and Child Hospital, Accra, GHA

**Keywords:** epidemiology, gastrointestinal complications, mortality, opioids, public health surveillance

## Abstract

Background

The opioid crisis has evolved beyond overdose deaths to include complex medical complications. Gastrointestinal (GI) complications represent a significant but underexplored aspect of opioid-related mortality. This study aimed to characterize trends in opioid-related deaths with concurrent GI complications in the United States from 2010 to 2020.

Methods

We conducted a retrospective, population-based analysis using mortality data from the Centers for Disease Control and Prevention Wide-ranging Online Data for Epidemiologic Research (CDC WONDER) Multiple Cause of Death database and the Global Burden of Disease (GBD) Study 2021. Opioid-related deaths were identified using ICD-10 codes T40.0-T40.4 and T40.6 (opium, heroin, other opioids, methadone, and synthetic and unspecified narcotics) in combination with K25-K28 (peptic ulcers), K56.0-K56.7 (intestinal obstruction), K59.0 (constipation), and K92.0-K92.2 (GI hemorrhage). Background GI mortality rates were obtained from GBD. Crude and age-adjusted mortality rates were calculated, and trends were analyzed using descriptive statistics.

Results

From 2010 to 2020, opioid-related deaths with GI complications increased by 218.6%, from 22,040 deaths (7.15 per 100,000) to 70,222 deaths (21.31 per 100,000). Males experienced disproportionately higher mortality rates (16.31 per 100,000) compared to females (7.76 per 100,000). West Virginia demonstrated the highest mortality rate (37.85 per 100,000), followed by Ohio (23.48 per 100,000) and Maryland (23.19 per 100,000). The steepest increases occurred between 2015 and 2017, with an unprecedented surge in 2020.

Conclusions

Opioid-related deaths with concurrent GI complications represent a rapidly escalating public health crisis, with mortality rates more than tripling over the study decade. The disproportionate impact on males and concentration in Appalachian and Rust Belt states suggest that targeted interventions are urgently needed.

## Introduction

The United States opioid epidemic has emerged as one of the most devastating public health crises of the 21st century, with over 500,000 drug overdose deaths reported since 1999 [1]. While much attention has focused on acute overdose mortality, the complex medical complications associated with chronic opioid use represent an equally concerning but underexamined aspect of this crisis [2].

Gastrointestinal (GI) complications constitute a significant portion of opioid-related morbidity and mortality. The pathophysiology involves multiple mechanisms: direct opioid receptor-mediated effects on gut motility leading to severe constipation and intestinal obstruction [3], delayed gastric emptying predisposing to aspiration [4], and concurrent substance use patterns that increase risks for peptic ulcer disease and GI hemorrhage [5]. Additionally, the social determinants affecting individuals with opioid use disorder, including delayed healthcare access and polydrug use, may exacerbate these complications [6].

Despite growing recognition of these complications in clinical practice, population-level surveillance of opioid-related deaths with concurrent GI pathology remains limited. Previous studies have primarily focused on emergency department presentations or hospitalization data [7,8], but mortality surveillance specifically capturing this intersection has been sparse.

The objective of this study was to characterize national trends in opioid-related deaths with concurrent GI complications in the United States from 2010 to 2020, examining temporal patterns, demographic disparities, and geographic distribution to inform targeted public health interventions.

## Materials and methods

Study design and data sources

We conducted a retrospective, population-based study using data from the Centers for Disease Control and Prevention Wide-ranging Online Data for Epidemiologic Research (CDC WONDER) Multiple Cause of Death database and the Global Burden of Disease (GBD) Study 2021. The study period spanned from 2010 to 2020, capturing the evolution of the opioid crisis through multiple phases, including prescription opioid escalation, heroin resurgence, and the fentanyl epidemic.

Case definition

Opioid-related deaths with concurrent GI complications were identified using ICD-10 codes T40.0-T40.4 and T40.6, which encompass deaths involving opium, heroin, natural and semi-synthetic opioids, methadone, synthetic narcotics, and other/unspecified narcotics, with concurrent documentation of K25-K28 (peptic ulcers), K56.0-K56.7 (intestinal obstruction), K59.0 (constipation), and K92.0-K92.2 (GI hemorrhage). This case definition captured deaths where both opioid involvement and GI pathology were documented as contributing factors.

Background mortality context

Background GI mortality rates were obtained from GBD 2021 data for peptic ulcer disease, paralytic ileus/intestinal obstruction, stomach cancer, colon cancer, and cirrhosis to provide epidemiological context for the observed trends.

Statistical analysis

Crude and age-adjusted mortality rates were calculated using U.S. Census population denominators. Annual trends were analyzed using descriptive statistics, with percentage changes calculated between 2010 and 2020. Demographic analyses were stratified by sex, and geographic analyses examined state-level variations in mortality rates. All analyses were conducted using standard epidemiological methods for mortality surveillance data.

Ethics statement

This study utilized de-identified, publicly available mortality data and was deemed exempt from IRB review; however, as per standard ethical practices, human consent was not required. No individual-level identifiable data were used.

## Results

From 2010 to 2020, opioid-related deaths with concurrent GI complications demonstrated a dramatic escalation, increasing by 218.6% from 22,040 deaths (crude rate: 7.15 per 100,000 population) in 2010 to 70,222 deaths (crude rate: 21.31 per 100,000 population) in 2020 (Table 1). The total mortality burden over the decade reached 421,523 deaths.

**Table 1:** Annual Opioid-Related Deaths With Gastrointestinal Complications, United States, 2010-2020 Total 2010-2020: 421,523 deaths

Year	Deaths (n)	Population	Crude Rate (per 100,000)
2010	22,040	308,341,000	7.15
2011	23,747	310,689,000	7.64
2012	24,098	313,336,000	7.69
2013	26,009	315,361,000	8.25
2014	29,650	318,386,000	9.30
2015	34,162	321,419,000	10.63
2016	43,491	323,128,000	13.46
2017	48,861	325,720,000	15.00
2018	48,065	327,167,000	14.69
2019	51,178	328,330,000	15.59
2020	70,222	329,485,000	21.31

The temporal pattern revealed distinct phases of acceleration. Initial increases from 2010 to 2014 were relatively modest, with annual death counts rising from 22,040 to 29,650. However, the period from 2015 to 2017 showed marked acceleration, with deaths increasing from 34,162 to 48,861. Following a brief plateau in 2018, mortality resumed its upward trajectory in 2019 and surged dramatically in 2020, representing the largest single-year increase observed during the study period.

Males experienced disproportionately higher mortality throughout the study period, with a total of 282,735 deaths (crude rate: 16.31 per 100,000) compared to 138,788 deaths among females (crude rate: 7.76 per 100,000) (Table 2). This represents a male-to-female mortality ratio of approximately 2.1:1, which remained consistent across the study decade.

**Table 2:** Opioid-Related Deaths With Gastrointestinal Complications by Sex, United States, 2010-2020

Sex	Total Deaths (n)	Population (2010-2020)	Crude Rate (per 100,000)	Ratio
Male	282,735	1,734,112,000	16.31	2.1:1
Female	138,788	1,788,549,000	7.76	-
Total	421,523	3,522,661,000	11.97	-

Ohio led the nation with 29,994 total deaths (crude rate: 23.48 per 100,000), followed by California with 28,399 deaths (crude rate: 6.66 per 100,000) and Florida with 28,237 deaths (crude rate: 12.66 per 100,000) (Table 3). New York and Pennsylvania rounded out the top five states by absolute death counts.

**Table 3:** Top 10 States by Total Opioid-Related Deaths With Gastrointestinal Complications, 2010-2020

Rank	State	Total Deaths (n)	Crude Rate (per 100,000)
1	Ohio	29,994	23.48
2	California	28,399	6.66
3	Florida	28,237	12.66
4	New York	26,634	12.36
5	Pennsylvania	20,474	14.57
6	Illinois	18,697	13.28
7	Massachusetts	15,687	21.09
8	Michigan	15,476	14.17
9	Texas	15,424	5.12
10	North Carolina	15,293	13.82

When examining crude mortality rates, West Virginia demonstrated the highest burden at 37.85 per 100,000 population, nearly double the rate of the second-highest state (Table 4). Ohio (23.48 per 100,000), Maryland (23.19 per 100,000), the District of Columbia (22.72 per 100,000), and New Hampshire (22.29 per 100,000) comprised the remaining top five jurisdictions by rate.

**Table 4:** Top 10 States by Crude Mortality Rate, Opioid-Related Deaths With Gastrointestinal Complications, 2010-2020

Rank	State	Crude Rate (per 100,000)	Total Deaths (n)
1	West Virginia	37.85	7,623
2	Ohio	23.48	29,994
3	Maryland	23.19	15,217
4	District of Columbia	22.72	1,592
5	New Hampshire	22.29	3,276
6	Delaware	21.90	2,271
7	Kentucky	21.44	10,427
8	Rhode Island	21.28	2,470
9	Massachusetts	21.09	15,687
10	Connecticut	18.99	7,481

**Figure 1:**
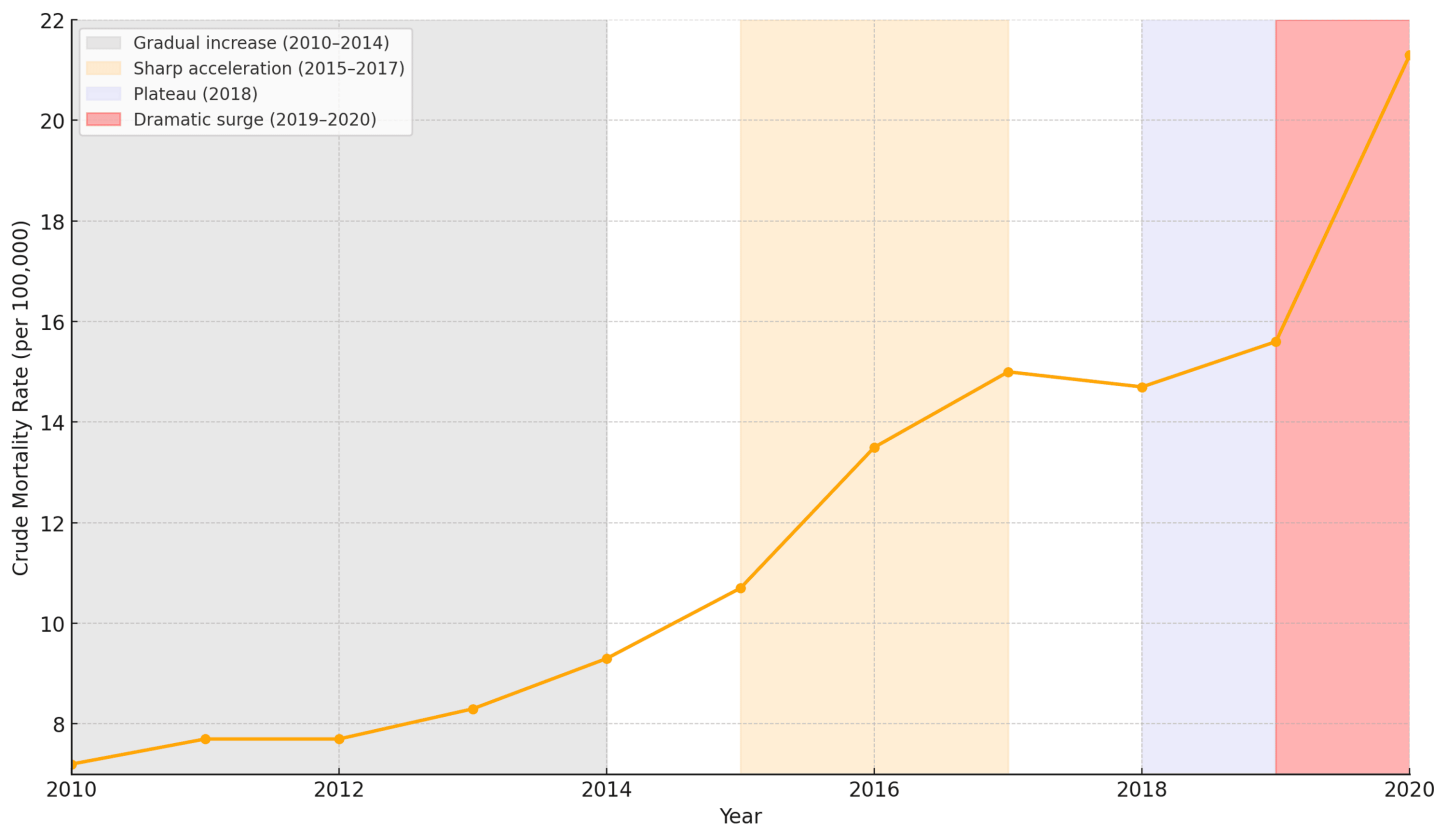
Temporal Trends in Opioid-Related Deaths With Gastrointestinal Complications, United States, 2010-2020 The figure illustrates crude mortality rates (deaths per 100,000 population) from 2010 to 2020. The trend includes a gradual increase from 2010 to 2014 (gray-shaded area), a sharp acceleration from 2015 to 2017 (yellow-shaded area), a plateau in 2018 (blue-shaded area), and a dramatic surge in 2020 (red-shaded area). These phases reflect the evolving impact of the opioid crisis and its gastrointestinal complications over the decade.

Clear geographic clustering emerged, with the highest rates concentrated in the Appalachian region (West Virginia, Kentucky, Ohio) and parts of the Northeast corridor (Maryland, Massachusetts, New Hampshire, Rhode Island, Connecticut). This distribution largely aligned with established patterns of the broader opioid crisis but showed particular concentration in economically distressed regions. The temporal evolution of the crisis is illustrated in Figure 1, which demonstrates the distinct phases of escalation from 2010 to 2020.

Comparison with background GI mortality from the GBD data revealed that opioid-related GI deaths represented a substantial and rapidly growing component of overall GI mortality. In 2020, the observed rate of 21.31 per 100,000 for opioid-related GI deaths exceeded background rates for peptic ulcer disease (2.96 per 100,000) and approached rates for major GI malignancies.

## Discussion

This study documents an unprecedented escalation in opioid-related deaths with concurrent GI complications, with mortality rates more than tripling from 2010 to 2020. The 218.6% increase represents one of the steepest mortality trends observed in modern U.S. public health surveillance, underscoring the multifaceted nature of the opioid crisis beyond acute overdose deaths.

The observed temporal patterns align with known phases of the opioid epidemic. The acceleration from 2015 to 2017 corresponds with the emergence of illicitly manufactured fentanyl in drug supplies [9], while the 2020 surge likely reflects the compounding effects of the COVID-19 pandemic on vulnerable populations [10]. The consistency of these patterns with broader opioid mortality trends suggests that GI complications represent an integral component of opioid-related deaths rather than an isolated phenomenon.

The persistent 2:1 male predominance likely reflects multiple factors, including higher rates of opioid use disorder among males [11], occupational exposures contributing to initial opioid use [12], and potentially delayed healthcare-seeking behavior leading to progression of GI complications [13]. This demographic pattern has important implications for targeted prevention and treatment strategies.

The concentration of the highest mortality rates in West Virginia, Ohio, and other Appalachian states reflects the complex intersection of economic distress, healthcare access limitations, and prescription opioid oversupply that has characterized this region's experience with the opioid crisis [14]. The elevated rates in these areas, often exceeding national averages by 200-300%, suggest that one-size-fits-all approaches may be insufficient and that regionally tailored interventions are necessary.

The magnitude of opioid-related GI mortality likely reflects several interconnected mechanisms. Direct opioid effects on μ-opioid receptors in the enteric nervous system can cause severe constipation progressing to intestinal obstruction [15]. Concurrent use of alcohol or non-steroidal anti-inflammatory drugs, common among individuals with opioid use disorder, substantially increases risks for peptic ulcer disease and GI hemorrhage [16]. Additionally, the social determinants affecting this population, including housing instability and limited healthcare access, may delay the recognition and treatment of emerging complications [17].

These findings have several important implications for public health policy and clinical practice. First, they underscore the need for expanded surveillance systems that capture the full spectrum of opioid-related mortality beyond acute overdose deaths. Second, they highlight the importance of integrated treatment approaches that address both addiction and medical comorbidities. Third, they highlight the critical need for targeted interventions in the states with the highest burden and specific demographic groups.

Limitations

Several limitations warrant consideration. First, our case definition relied on the accuracy of ICD-10 coding, which may vary across jurisdictions and over time. Some opioid-related deaths with GI complications may have been missed if not properly coded, suggesting that our estimates may be conservative. Second, the analysis focused on mortality rather than morbidity, which may have potentially underestimated the full burden of these complications. Third, we could not assess specific opioid types or routes of administration, which may have different risk profiles for GI complications.

Future directions

Future research should examine age-stratified patterns, as our analysis focused on crude rates. Additionally, investigation of specific GI complication types and their temporal trends would provide more granular insights for targeted interventions. Analysis of healthcare utilization patterns preceding these deaths could inform prevention strategies, and examination of state-level policy correlates might identify effective intervention approaches.

## Conclusions

Opioid-related deaths with concurrent GI complications represent a rapidly escalating public health crisis that has received insufficient attention despite its substantial and growing mortality burden. The more than threefold increase in mortality rates from 2010 to 2020, with particularly severe impacts on males and residents of Appalachian states, demands an urgent and targeted public health response. These findings underscore the complex, multisystem nature of the opioid crisis and highlight the critical need for integrated approaches that address both addiction treatment and management of medical complications. The geographic concentration of the highest mortality rates suggests that regionally tailored interventions, particularly in West Virginia, Ohio, and surrounding areas, should be prioritized.

As the opioid crisis continues to evolve, comprehensive surveillance systems must expand beyond acute overdose deaths to capture the full spectrum of opioid-related mortality. Only through such comprehensive approaches can public health systems adequately respond to and ultimately address this multifaceted crisis.
